# Hematochezia due to arteriovenous malformation of the mesoappendix: a rare case report

**DOI:** 10.1093/jscr/rjad164

**Published:** 2023-04-12

**Authors:** Tuan Anh Nguyen, Hiep Van Pham, Thang Manh Tran

**Affiliations:** Department of Digestive Surgery, Institute of Digestive Surgery, 108 Military Central Hospital, Hanoi, Vietnam; Department of Digestive Surgery, Institute of Digestive Surgery, 108 Military Central Hospital, Hanoi, Vietnam; College of Health Sciences, VinUniversity, Hanoi, Vietnam

**Keywords:** Arteriovenous malformations, AVMs, Laparoscopic appendectomy, Mesoappendix, Lower gastrointestinal bleeding

## Abstract

An arteriovenous malformation (AVM) of the mesoappendix is a very rare clinical entity. The clinical features, diagnosis and management of an AVM of the mesoappendix have yet to be sufficiently explained since reports about it are scarce. We report a 57-year-old man presented with hematochezia for 2 weeks. Upper and lower endoscopic could not find the source of bleeding. Abdominal contrast-enhanced computed tomography revealed an AVM of the mesoappendix. A laparoscopic appendectomy was performed, and he had a resolution of his symptoms. Intraoperative findings and pathological results confirmed the diagnosis of AVM of the mesoappendix.

## INTRODUCTION

Acute gastrointestinal bleeding is a major cause of hospitalization in the clinical setting [1], and lower gastrointestinal bleeding (LGIB) accounts for 30–40% [2]. LGIB typically presents with painless hematochezia. Nevertheless, up to 15% of patients with hematochezia had an upper source to account for the bleeding [3]. The main sources of LGIB are diverticulosis, angiodysplasia, hemorrhoids, colitis or neoplasms [4]. However, LGIB secondary to appendix bleeding is very rare. The recommended treatment of appendiceal hemorrhage is surgical, with reports of appendectomy, ileocecectomy, partial cecectomy or hemicolectomy [5].

An arteriovenous malformation (AVM) of the mesoappendix is extremely rare, and reports of AVM of the mesoappendix are limited; we cannot find any other report except one report about AVM of the appendix on Pubmed [6]. Thus, their clinical features, diagnosis and adequate management have not been explained sufficiently. In this article, we report a case of isolated AVM of the mesoappendix, which was successfully managed by laparoscopic appendectomy.

## CASE REPORT

**Figure 1:**
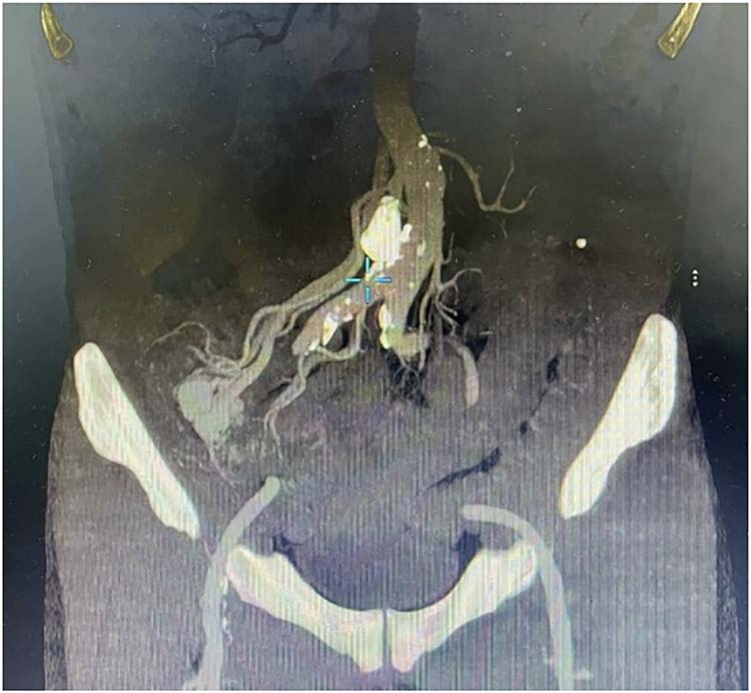
Contrast-enhanced abdominal CT showed a thickened appendix without signs of inflammation and images of vascular proliferation and dilated veins around the appendix.

**Figure 2:**
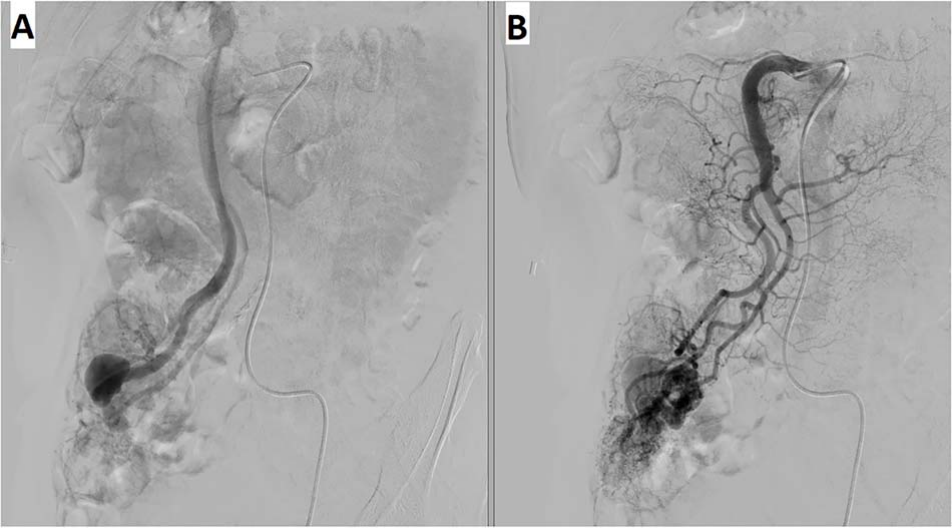
Angiography revealed (**A**) vascular tangle with (**B**) early venous filling in the appendicular artery and vein.

A 57-year-old male patient with a history of hypertension was referred to our hospital for multiple episodes of painless bloody stools for 2 weeks. No gastrointestinal symptoms were observed, such as abdominal pain, nausea or vomiting. A digital rectal examination showed bright red blood. On admission, laboratory evaluation revealed mild anemia with hemoglobin of 103 g/dL. Other than that, there were no other abnormalities. Lower and upper gastrointestinal endoscopy was performed, but the cause of the bleeding could not be identified. Due to the signs and symptoms of ongoing bleeding, we decided to perform imaging tests to make a diagnosis. Abdominal contrast-enhanced computed tomography (CT) revealed a thickened appendix (17 mm in diameter and wall thickening 6.4 mm) without signs of inflammation and images of vascular proliferation and dilated vein around the appendix (Fig. 1). An angiography was performed and demonstrated the AVM of the appendicular artery and vein (Fig. 2).

**Figure 3:**
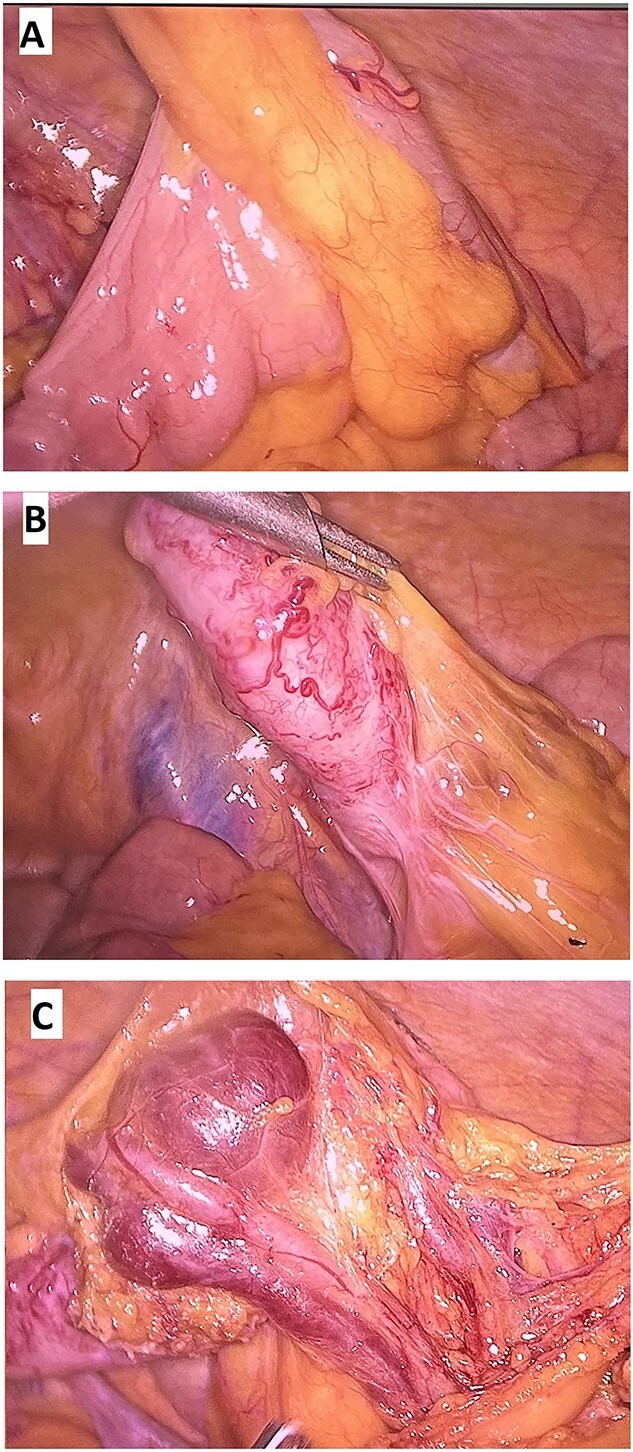
(**A**, **B**) Mesentary of the ileum was normal; (**C**) Thickened appendix with the AVM in the appendix mesentery.

Based on that information, the patient was diagnosed with LGIB due to AVM of the appendix and underwent a laparoscopic appendectomy. Intraoperative findings were consistent with imaging studies (Fig. 3). The mesentery of the ileum was normal, and the appendix was enlarged and thickened (⁓15 mm in diameter). At the appendix mesentery, we saw the AVM of the appendicular artery and vein with the dilation of the vein (⁓30 mm in diameter). After dissecting the mesoappendix, we continued to dissect along the vein further; those veins drained to the superior mesenteric vein, and no more AVM were observed. We clipped those vessels by ligation clips and divided them by LigaSure. The appendiceal base was divided using Endo gastrointestinal anastomosis Stapler.

**Figure 4:**
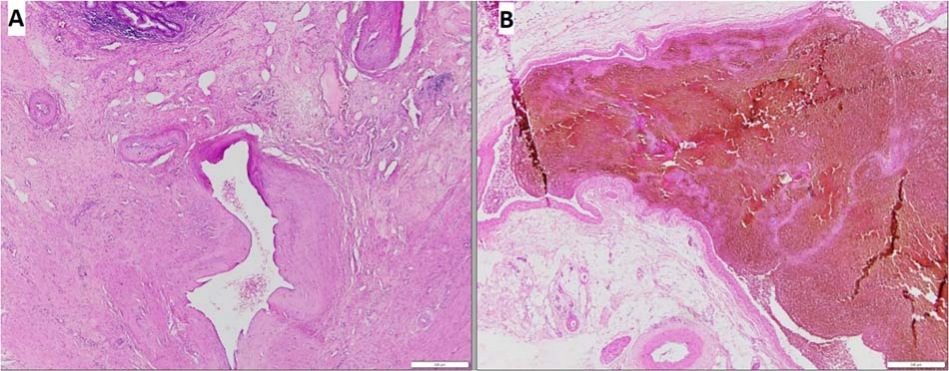
The hematoxylin and eosin stain demonstrated an AVM of the mesoappendix: (**A**) Thick and thin walled vessels in the submucosal layer and the muscular layer, (**B**) On high power field, large vessels with dilated lumens.

Histopathological results confirmed that this was an AVM of the mesoappendix (Fig. 4). The patient was discharged on postoperative day 2. At the 4-month follow-up, he was doing well and denied any signs and symptoms of re-bleeding.

## DISCUSSION

LGIB is a common problem in the clinical setting. Major causes of LGIB include diverticular disease, anorectal disease, ischemia and neoplasia. Other less common causes include infectious colitis, postpolypectomy, inflammatory bowel disease, angiodysplasia, radiation colitis or proctitis. In up to 25% of patients with LGIB, the source of bleeding is never accurately identified [7]. However, bleeding from the appendix is an infrequent cause of LGIB. This is even though the same factors that are responsible for LGIB (such as inflammation, diverticulosis, tumor or damage to the appendix mucosa) can also cause appendiceal bleeding [8–11]; however, the source from the appendix may not be identified. Clinicians should raise the suspicion of appendix in patients with difficult-to-diagnose LGIB. In our patient, both upper and lower endoscopy could not find the culprit of the hemorrhage.

AVM in the mesoappendix is a very rare clinical entity and has been reported by some authors where AVM is the etiology of appendicitis [12, 13]. Contrast-enhanced CT and angiography are good choices for looking for vascular malformation. CT scan with IV contrast not only helps to identify vascular lesions but also helps to rule out other bowel wall lesions. Mesenteric angiography is effective in diagnosis as well as intervention. However, the appendicular artery angiographic interventions were relatively contraindicated because of the high risk of ischemia of the appendix and appendicitis [14]. For that reason, surgical resection is considered as the standard of management. Intraoperatively, this is important to identify and remove all AVM lesions to avoid new lesions that may develop after incomplete resection [15]. In this case, we performed a laparoscopic appendectomy; during the operation, we also dissected the appendicular vein up to the level of the superior mesenteric vein to rule out any other AVM.

In conclusion, LGIB secondary to the AVM in the mesoappendix is very rare, but we should always consider it in mind to consider in LGIB patients when we cannot find the source. A laparoscopic approach is effective and safe for managing AVM in the mesoappendix.

## Data Availability

Data available on request due to privacy/ethical restrictions. The data that support the findings of this study are available on request from the corresponding author.
